# Isolation, NMR Spectral Analysis and Hydrolysis Studies of a Hepta Pyranosyl Diterpene Glycoside from *Stevia rebaudiana* Bertoni

**DOI:** 10.3390/biom3040733

**Published:** 2013-09-30

**Authors:** Venkata Sai Prakash Chaturvedula, Steven Chen, Oliver Yu, Guohong Mao

**Affiliations:** Natural Ingredient Development, Blue California, 30111 Tomas, Rancho Santa Margarita, CA 92688, USA; E-Mails: steven@bluecal-ingredients.com (S.C.); oliver@bluecal-ingredients.com (O.Y.); guohongmao@yahoo.com (G.M.)

**Keywords:** *Stevia rebaudiana*, diterpene glycoside, isolation, structure elucidation, spectral data, hydrolysis studies

## Abstract

From the commercial extract of the leaves of *Stevia rebaudiana* Bertoni, a minor steviol glycoside, 13-[(2-*O*-β-d-glucopyranosyl-3-*O*-β-d-glucopyranosyl-β-d-glucopyranosyl)oxy] *ent*-kaur-16-en-19-oic acid-[(2-*O*-(3-*O*-β-d-glucopyranosyl-α-l-rhamnopyranosyl)-3-*O*-β-d-glucopyranosyl-β-d-glucopyranosyl) ester] (**1**); also known as rebaudioside O having seven sugar units has been isolated. Its structural characterization has been achieved by the extensive 1D (^1^H and ^13^C), and 2D NMR (COSY, HMQC, HMBC) as well as mass spectral data. Further, hydrolysis studies were performed on rebaudioside O using acid and enzymatic methods to identify aglycone and sugar residues in its structure as well as their configurations.

## 1. Introduction

*Stevia rebaudiana* (Bertoni) Bertoni is a perennial shrub of the Asteraceae (Compositae) family native to Brazil and Paraguay, which is often referred to as “The sweet herb of Paraguay”. Currently, *S. rebaudiana* is being grown commercially in a number of countries, particularly in China, Japan, Taiwan, Korea, Thailand and Indonesia [1,2]. Extracts of the leaves of *S. rebaudiana* have been used for decades to sweeten food and beverages in Japan, South America and China. The major constituents in the leaves of *S. rebaudiana* are the potently sweet glycosides namely steviolbioside, stevioside, rebaudiosides A and E, dulcoside A and rubusoside; which are glycosides of the diterpene steviol, *ent*-13-hydroxykaur-16-en-19-oic acid [3,4]. These compounds are also known as Stevia sweeteners. 

**Figure 1:**
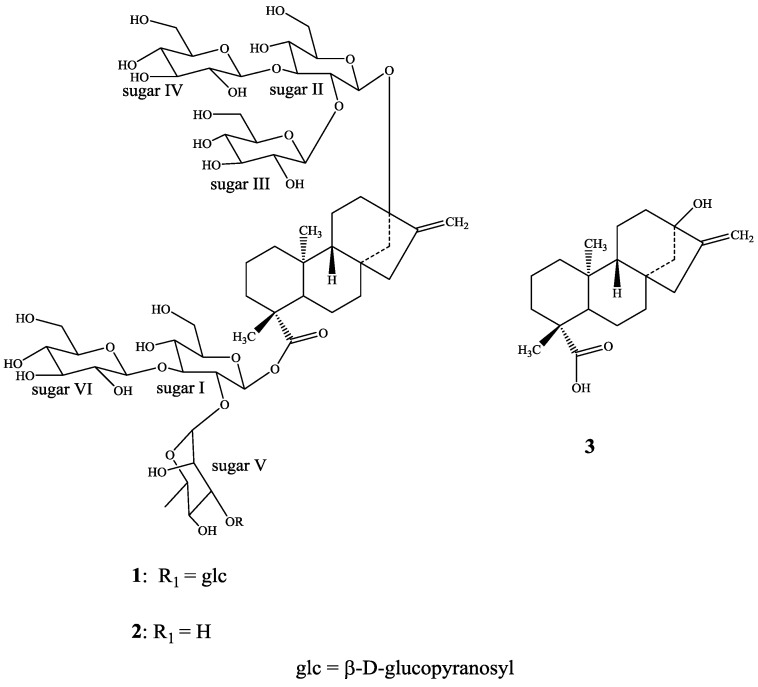
Structure of rebaudioside O (**1**), rebaudioside N (**2**), and steviol (**3**).

Recently, Ohta *et al*. have reported several minor steviol glycosides including rebaudioside O (**1**) from *S. rebaudiana* Morita, which was developed as a cultivar by selective breeding of *S. rebaudiana* Bertoni. However, they have not reported isolation or complete spectral assignments of pure rebaudioside O [5]. As a part of our research related to the discovery of natural sweeteners and sweetener enhancers, we are herewith describing the isolation, characterization and complete ^1^H- and ^13^C-NMR spectral assignments for the diterpene glycoside 13-[(2-*O*-β-d-glucopyranosyl-3-*O*-β-d-glucopyranosyl-β-d-glucopyranosyl)oxy] *ent*-kaur-16-en-19-oic acid-[(2-*O*-(3-*O*-β-d-glucopyranosyl-α-l-rhamnopyranosyl)-3-*O*-β-d-glucopyranosyl-β-d-glucopyranosyl) ester] (**1**), also known as rebaudioside O (Figure 1) isolated from the commercial extract of *S. rebaudiana* Bertoni. The complete NMR assignments were achieved on the basis of 1D (^1^H and ^13^C) and 2D (COSY, HMQC and HMBC) NMR as well as high resolution mass spectroscopic data. Acid and enzymatic hydrolysis studies on compound **1** were carried out to identify aglycone and sugar residues.

## 2. Results and Discussion

Compound **1** was isolated as a white powder and its molecular formula has been deduced as C_62_H_100_O_37_ on the basis of its positive ESI TOF mass spectrum which showed [M+Na]^+^ ion at *m/z* 1,459.5854; this composition was supported by ^13^C-NMR spectral data. The ^1^H-NMR spectrum of **1** showed the presence of two methyl signal resonating at δ 1.17 and 1.54 as singlets, two olefinic protons at δ 5.05 and 5.70 as singlets corresponding to an exocyclic double bond, nine methylene and two methine protons between δ 0.69–2.56, characteristic for the *ent*-kaurane diterpenoids isolated earlier from the genus *Stevia* [6,7,8,9]. The basic skeleton of *ent-*kaurane diterpenoids was supported by COSY (H-1/H-2; H-2/H-3; H-5/H-6; H-6/H-7; H-9/H-11; H-11/H-12) and HMBC (H-1/C-2, C-10; H-3/C-1, C-2, C-4, C-5, C-18, C-19; H-5/C-4, C-6, C-7, C-9, C-10, C-18, C-19, C-20; H-9/C-8, C-10, C-11, C-12, C-14, C-15; H-14/C-8, C-9, C-13, C-15, C-16 and H-17/C-13, C-15, C-16) correlations. Also, the ^1^H NMR spectrum of **1** showed the presence of seven sugar units in its structure by the presence of the anomeric protons resonating at δ 4.97, 5.04, 5.38, 5.49, 5.69, 6.16, and 6.21; which was further supported by the MS/MS spectrum of **1** in the positive ESI mode which showed the fragment ions at *m/z* 1,297, 1,135, 973, 827, 665, 503, and 341, suggesting the presence of six hexose and one deoxyhexose units in its structure. Acid hydrolysis of **1** with 5% H_2_SO_4_ afforded the sugars d-glucose and l-rhamnose, which were identified by direct comparison with authentic samples by TLC [10,11,12]. Further, the identification of sugars present in **1 ** and their configurations were achieved by preparing their thiocarbamoyl-thiazolidine carboxylate derivatives with l-cysteine methyl ester and *O*-tolyl isothiocyanate, and in comparison of their retention times with the standard sugars as described in the literature; suggesting the sugar moieties present as β-d-glucopyranosyl and α-l-rhamnopyranosyl units [13]. Enzymatic hydrolysis of **1** furnished an aglycone which was identified as steviol (**3**) by comparison of ^1^H-NMR and co-TLC with standard compound [14]. The large coupling constants observed for the six anomeric protons of the glucose moieties at δ 4.97 (d, *J* = 7.8 Hz), 5.04 (d, *J* = 7.6 Hz), 5.38 (d, *J* = 8.1 Hz), 5.49 (d, *J* = 8.1 Hz), 5.69 (d, *J* = 8.4 Hz), and 6.16 (d, *J* = 8.1 Hz), suggested their β-orientation as reported for steviol glycosides [6,7,8,9]. The seventh anomeric sugar corresponding to that of l-rhamnosyl unit was identified as a doublet at δ 6.21 (*J* = 1.7 Hz) suggesting its α-orientation [5]. The ^1^H- and ^13^C-NMR values for all the carbons in **1** were assigned on the basis of COSY, HSQC and HMBC correlations (Table 1). 

**Figure 2:**
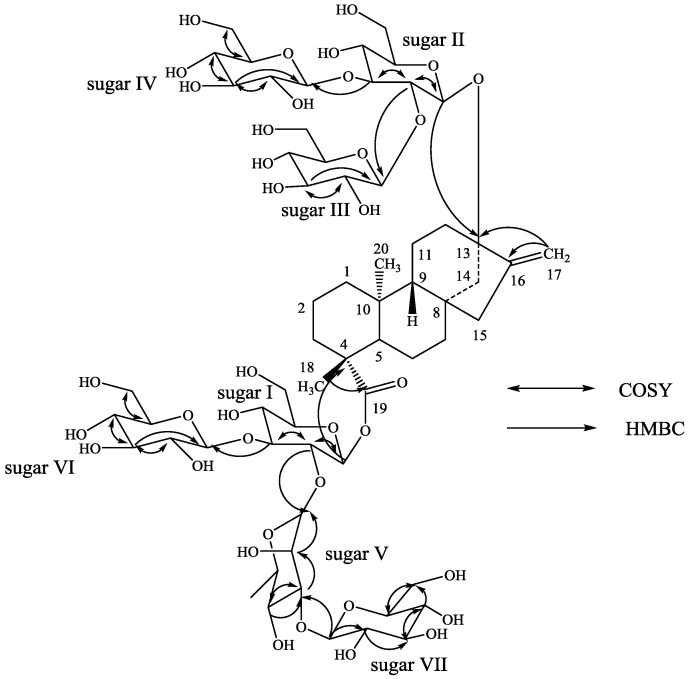
Key COSY and HMBC correlations of rebaudioside O (**1**).

Based on the results from NMR spectral data and hydrolysis experiments of **1**, it was concluded that there are six β-d-glucosyl units and an α-l-rhamnosyl unit in its structure connected to the aglycone steviol. A close comparison of the ^1^H- and ^13^C-NMR values of **1** with rebaudioside N (**2**) [5] suggested the presence of a steviol aglycone moiety with a Glcβ1–2(Glcβ1–3)-Glcβ1 unit at C-13 in the form of ether linkage and another Rhaα1–2(Glcβ1–3)-Glcβ1 unit at C-19 position in the form of an ester linkage, leaving the assignment of the additional β-d-glucosyl unit. The downfield shift for both the ^1^H and ^13^C chemical shifts at C-3′ of sugar V of the α-l-rhamnosyl moiety suggested that the additional β-d-glucosyl unit has been attached at this position. This was confirmed by the key HMBC correlations: H-3′′′′′/C-1′, C-3′, C-1′′′′′′′, H-1′′′′′′′/C-2′′′′′, C-2′′′′′′′, C-3′′′′′′′ and H-2′′′′′′′/C-1′′′′′′′, C-3′′′′′′′. Based on the results from chemical and spectral studies, **1** was assigned as 13-[(2-*O*-β-d-glucopyranosyl-3-*O*-β-d-glucopyranosyl-β-d-glucopyranosyl)oxy] *ent*-kaur-16-en-19-oic acid-[(2-*O*-(3-*O*-β-d-glucopyranosyl-α-l-rhamnopyranosyl)-3-*O*-β-d-glucopyranosyl-β-d-glucopyranosyl) ester]. The structure was further supported by the key COSY and HMBC correlations as shown in Figure 2.

**Table 1:** ^1^H- and ^13^C-NMR spectral data (chemical shifts and coupling constants) for rebaudioside O (**1**) in d5-pyridine (C_5_D_5_N) ^a–c^.

Position	^1^H-NMR	^13^C-NMR
1	0.69 br t (12.4), 1.62 m	40.4
2	1.35 m, 2.13 m	19.6
3	1.11 br t (11.6), 2.52 d (12.4)	38.5
4	---	44.9
5	0.96 d (12.6)	58.9
6	1.88 m, 2.10 m	22.7
7	1.26 br t (11.4), 1.35 br d (11.4)	42.4
8	---	41.3
9	0.87 br s	54.6
10	---	40.4
11	1.62 m	21.3
12	1.87 m, 2.14 m	38.3
13	---	89.7
14	1.74 d (10.6), 2.56 d (11.4)	45.2
15	2.01 m, 2.16 m	48.6
16	---	154.7
17	5.05 s, 5.70 s	105.5
18	1.54 s	30.1
19	---	176.0
20	1.17 s	17.7
1′	6.16 d (8.1)	94.2
2′	4.52 m	81.3
3′	4.32 m	88.9
4′	4.28 m	70.5
5′	3.91 m	78.1
6′	4.12 m, 4.34 m	62.6
1′′	4.97 d (7.8)	98.7
2′′	4.42 m	79.2
3′′	4.36 m	87.4
4′′	4.22 m	69.7
5′′	3.62 m	77.2
6′′	4.14 m, 4.36 m	62.9
1′′′	5.69 d (8.4)	105.4
2′′′	4.16 m	76.8
3′′′	4.32 m	78.8
4′′′	4.22 m	72.3
5′′′	3.86 m	79.0
6′′′	4.38 m, 4.56 m	63.9
1′′′′	5.38 d (8.1)	105.2
2′′′′	4.06 m	75.7
3′′′′	4.36 m	79.2
4′′′′	4.24 m	72.1
5′′′′	4.16 m	79.3
6′′′′	4.22 m, 4.54 d (11)	63.0
1′′′′′	6.21 d (1.7)	102.3
2′′′′′	4.80 br s	73.0
3′′′′′	4.52 m	84.5
4′′′′′	4.42 m	72.3
5′′′′′	4.46 m	70.4
6′′′′′	1.69 d (6.7)	19.6
1′′′′′′	5.04 d (7.6)	105.1
2′′′′′′	4.08 m	76.6
3′′′′′′	4.26 m	79.2
4′′′′′′	4.16 m	72.9
5′′′′′′	4.24 m	78.9
6′′′′′′	41.6 m, 4.34 m	62.9
1′′′′′′′	5.49 d (8.1)	107.5
2′′′′′′′	4.04 m	77.1
3′′′′′′′	4.32 m	79.2
4′′′′′′′	4.22 m	72.3
5′′′′′′′	3.92 m	79.1
6′′′′′′′	4.38 m, 4.56 m	63.3

^a^ assignments made on the basis of COSY, HSQC and HMBC correlations; ^b^ Chemical shift values are in δ (ppm); ^c ^Coupling constants are in Hz.

This is the first report of the isolation of rebaudioside O (**1**) from *S. rebaudiana* Bertoni though it has been reported earlier from *S. rebaudiana* Morita [5]. Further, partial NMR spectral data has been reported earlier for rebaudioside O (**1**) [5], whereas we are herewith reporting its complete ^1^H- and ^13^C-NMR spectral assignments based on 1D (^1^H and ^13^C) and 2D (COSY, HMQC and HMBC) NMR as well as high resolution mass spectroscopic data which was supported by enzymatic and acid hydrolysis studies.

## 3. Experimental

### 3.1. General

HPLC analysis was performed using a Dionex UPLC ultimate 3000 system (Sunnyvale, CA, USA), including a quaternary pump, a temperature controlled column compartment, an auto sampler and a UV absorbance detector. Phenomenex Luna NH_2_ with guard column, 150 × 3.0 mm, 3 µm (100A) were used for the characterization of rebaudioside O (**1**). Analytical HPLC was carried out with a Waters 600E multisolvent delivery system using a Phenomenex Luna C18 (150 × 4.6 mm, 5 µm) column. NMR spectra were acquired on a Varian INOVA 600 MHz instrument with a 5 mm HCN probe using standard pulse sequences. The NMR spectra were performed in C_5_D_5_N; chemical shifts are given in δ (ppm), and coupling constants are reported in Hz. The spectral data was referenced to the residual solvent signal (δ_H_ 7.19, and δ_C_ 123.5 for pyridine-d_5_). IR spectral data was acquired using a Perkin Elmer 400 Fourier Transform Infrared (FT-IR) Spectrometer with Universal attenuated total reflectance (UATR) polarization accessory. MS and MS/MS data were generated with a Thermo LTQ-FTMS mass spectrometer (100,000 resolutions) equipped with a Nano spray ionization source. Samples were diluted with methanol and introduced via infusion using the onboard syringe pump. 

### 3.2. Isolation

Compound **1** was purified by repeated isocratic elution (72% acetonitrile in water) of the commercial extract of *S. rebaudiana* using Dionex UPLC ultimate 3000 system with Phenomenex Luna NH_2_ guard column. We then collected the peak eluting at *t_R_* 9.82 min; and dried the corresponding solution under nitrogen yielded **1**.

*13-[(2-O-β-D-glucopyranosyl-3-O-β-D-glucopyranosyl-β-D-glucopyranosyl)oxy] ent-kaur-16-en-19-oic acid-[(2-O-(3-O-β-D-glucopyranosyl-α-L-rhamnopyranosyl)-3-O-β-D-glucopyranosyl-β-D-glucopyranosyl) ester] (Rebaudioside O, **1**).* White powder; IR ν_max_: 3335, 2942, 1728, 1058, 918 cm^−1^; ^1^H-NMR (600 MHz, C_5_D_5_N, δ ppm) and ^13^C-NMR (150 MHz, C_5_D_5_N, δ ppm) spectroscopic data see Table 1; HRMS (M+H)^+^ *m/z* 1459.5854 (calcd. for C_62_H_100_O_37_Na: 1459.5841).

*Acid hydrolysis of compound **1**.* To a solution of compound **1** (5 mg) in MeOH (10 mL) was added 3 mL of 5% H_2_SO_4_ and the mixture was refluxed for 24 h. The reaction mixture was then neutralized with saturated sodium carbonate and extracted with ethyl acetate (EtOAc) (2 × 25 mL) to give an aqueous fraction containing sugars and an EtOAc fraction containing the aglycone part. The aqueous phase was concentrated and compared with standard sugars using the TLC systems EtOAc/*n*-butanol/water (2:7:1) and CH_2_Cl_2_/MeOH/water (10:6:1) [10,11,12]; the sugars were identified as d-glucose and l-rhamnose. 

*Determination of sugar configuration in **1**.* Compound **1** (1 mg) was hydrolyzed with 0.5 M HCl (2 mL) for 1.5 h. The reaction mixture was cooled to room temperature, passed through an Amberlite IRA400 column and the eluate was lyophilized. The residue was dissolved in pyridine (1 mL) and heated with l-cysteine methyl ester HCl (5 mg) at 60 °C for 1.5 h, and then *O*-tolyl isothiocyanate (25 uL) was added to the mixture and heated at 60 °C for an additional 1.5 h. The reaction mixture was analyzed by HPLC: column Phenomenex Luna C18, 150 × 4.6 mm (5 µ); Mobile phase: 25% acetonitrile in water with 0.2% TFA, Flow rate: 1 mL/min; UV detection at 250 nm. The sugars were identified as d-glucose (*t*R, 12.48 min) and l-rhamnose (*t*R, 21.58 min) [authentic samples, d-glucose (*t*R, 12.64) and l-glucose (*t*R, 11.36 min); d-rhamnose (*t*R, 12.02) and l-rhamnose (*t*R, 21.76 min)] [13].

*Enzymatic hydrolysis of compound **1**.* Compound **1** (1 mg) was dissolved in 10 mL of 0.1 M sodium acetate buffer, pH 4.5 and crude pectinase from *Aspergillus niger* (100 uL, Sigma-Aldrich, P2736) was added. The mixture was stirred at 50 °C for 72 h. The product precipitated out during the reaction and was filtered and then crystallized. The resulting product obtained from the hydrolysis of **1** was identified as steviol (**3**) by comparison of its co-TLC with standard compound and ^1^H NMR spectral data [14]. 

## 4. Conclusions

To the best of our knowledge, this is the first report of the isolation of rebaudioside O from *S. rebaudiana* Bertoni. Also, we are herewith reporting the complete ^1^H- and ^13^C-NMR spectral assignments for 13-[(2-*O*-β-d-glucopyranosyl-3-*O*-β-d-glucopyranosyl-β-d-glucopyranosyl)oxy] *ent*-kaur-16-en-19-oic acid-[(2-*O*-α-d-rhamnopyranosyl-3-*O*-β-d-glucopyranosyl-β-d-glucopyranosyl) ester], also known as rebaudioside O (**1**) on the basis of extensive 1D- and 2D-NMR as well as high resolution mass spectral data. Further, acid hydrolysis furnished two sugar units that were identified as β-d-glucose and α-l-rhamnose; and enzymatic hydrolysis furnished steviol.
